# Novel Big Data-Driven Machine Learning Models for Drug Discovery Application

**DOI:** 10.3390/molecules27030594

**Published:** 2022-01-18

**Authors:** Vishnu Sripriya Akondi, Vineetha Menon, Jerome Baudry, Jana Whittle

**Affiliations:** 1 Department of Computer Science, The University of Alabama in Huntsville, Huntsville, AL 35899, USA; va0017@uah.edu; 2 Department of Biological Sciences, The University of Alabama in Huntsville, Huntsville, AL 35899, USA; jerome.baudry@uah.edu (J.B.); jsw0013@uah.edu (J.W.)

**Keywords:** drug discovery, class imbalance, machine learning, protein conformation selecton, drug candidates, ADORA2A, OPRK1

## Abstract

Most contemporary drug discovery projects start with a ‘hit discovery’ phase where small chemicals are identified that have the capacity to interact, in a chemical sense, with a protein target involved in a given disease. To assist and accelerate this initial drug discovery process, ’virtual docking calculations’ are routinely performed, where computational models of proteins and computational models of small chemicals are evaluated for their capacities to bind together. In cutting-edge, contemporary implementations of this process, several conformations of protein targets are independently assayed in parallel ‘ensemble docking’ calculations. Some of these protein conformations, a minority of them, will be capable of binding many chemicals, while other protein conformations, the majority of them, will not be able to do so. This fact that only some of the conformations accessible to a protein will be ’selected’ by chemicals is known as ’conformational selection’ process in biology. This work describes a machine learning approach to characterize and identify the properties of protein conformations that will be selected (i.e., bind to) chemicals, and classified as potential binding drug candidates, unlike the remaining non-binding drug candidate protein conformations. This work also addresses the class imbalance problem through advanced machine learning techniques that maximize the prediction rate of potential protein molecular conformations for the test case proteins ADORA2A (Adenosine A2a Receptor) and OPRK1 (Opioid Receptor Kappa 1), and subsequently reduces the failure rates and hastens the drug discovery process.

## 1. Introduction

The core concept of any drug discovery application involves, in most cases, a protein biological target which binds with a chemical (known as ’ligand’) to achieve a biological function. The ligand is most often, although not always, a small organic chemical that binds to the protein target and modulates its function in a way that achieves a therapeutic benefit to the patient. Indeed, drug discovery can be inferred as the process of identification of small molecules that will bind selectively and safely to specific protein targets that are responsible for diseases.

The modern-day drug discovery and development timeline is a complex process that starts with protein target identification and ends with an FDA approval, and that takes an average of 12–15 years and costs more than $1 billion until the launch of the finished product. These time and cost issues are mostly due to (i) the time it takes in the early stage to identify drug candidates effective against a protein target, and (ii) the inflated failure rates of over 90% during later clinical trial stages, where the identified potential drug candidate proteins fail to succeed during various stages of developmental clinical trials. The prominent cause of these late-stage failures is that the potential drug candidates often have a tendency to bind off-target proteins, instead of only binding to their intended protein targets. This off-target drug binding can lead to a spectrum of adverse effects ranging from lack of potency and selectivity to more serious issues of drug toxicity [1].

A popular and efficient approach to identify in silico chemicals that have the potential to bind to specific proteins is called docking. In docking, computational models of chemicals are used together with computational models of the protein of interest to compute their respective binding free energies. These chemicals that are predicted to bind strongly to the protein of interest would be prioritized for further experimental testing. This approach has been historically used in many early-scale drug discovery projects. Thanks to the availability of supercomputers, this process is now beginning to be applied to the prediction of later-stage clinical trial outcomes, predicting not only of the chemicals of interest can bind to the protein of interest, but also if they can bind to several proteins known to be responsible for many clinical trial failures because of off-target binding [1].

A recent major development of this docking approach is the use of ensemble docking [2]. In ensemble-docking, more than one computational model of the protein target is used, combinatorially, to predicted the binding affinities of chemicals against the protein. The idea behind ensemble-docking is that, in the living cells, proteins are not static objects, but they undergo constant conformational dynamics that change their shapes to a significant extent at the atomistic level, and hence the binding affinities of chemicals. We have shown that in the many conformations that a protein can adopt, some, a small minority, will be much better at binding chemicals than others, confirming the chemical concept of conformational selection of protein conformations by their ligands [1]. In order to describe the range of conformations that the target proteins can adopt, various approaches such as molecular dynamics (MD) simulations Monte Carlo or Markov-State models are often employed [3]. However, the use of MD simulations to emulate the protein-ligand docking process has its own share of challenges beyond the knowledge of the structure of both the target protein and ligands [3]. In the context of ensemble docking, the most important challenge is how to identify, among a large number of conformational variations of a protein structure, the few ones that will end up being selected by the drugs for binding [1]. This work aims at addressing this important question by looking at physical and data properties of protein conformations that are associated with conformational selection. MD simulations do not provide data-driven insights into the identification of data-patterns such as the biophysical or chemical properties of ligand at-large that enables the selection of specific target protein conformations over the others. These problems highlight the need for advanced machine learning (ML) techniques that can analytically determine a precise binding with the least amount of time, cost expended with minimal pre-clinical lab trials and reduced failure rates at much early-stages in the drug discovery pipeline.

In literature, commonly used ML techniques based on characterizing protein conformational selection include root mean squared deviation (RMSD) clustering, Perron cluster analysis (PCCA+), and Markov-clustering (MCL) methods [4]. RMSD is a least squares approach that computes protein conformational RMSD values to generate clusters or protein conformational states based on a user-defined threshold. PCCA+ is a fuzzy clustering algorithm that performs clustering on the eigenvalues of the protein conformational transitions. MCL is a graph based clustering algorithm that uses the probability of a random walker to transition to a particular sequence of states. Strecker and Meyer performed clustering methods such as K-Means and hierarchical clustering (with average linkage) analysis on the cluster representatives to measure the docking performance in terms of binding pose prediction, screening utility and scoring accuracy [5]. Other mutual information based methods [6,7] and dimensionality reduction methods have also been proposed for information extraction [8].

However, the current ML methods do not address the caveats of statistically ill-conditioned and limited real-world data availability scenarios. This brings us to the class imbalance problem, where the detection of smaller sample-size potential drug-binding protein conformations is dominated by the larger population of non-drug-binding protein conformations. Consequently, most of the ML techniques employed for data-learning on such a biased population dataset will result in a high risk that the smaller population of potential drug candidate protein conformations (minority class samples) will be misclassified/ misidentified as the non-drug candidate protein conformations. In practice, this class imbalance problem could lead to serious effects where the researchers arrive at erroneous conclusions on the probable subset of proteins that will generate, from docking, a correct and useful list of chemicals predicted to bind to the protein targets, thereby drastically reducing the impact and reliability of analytical solutions for drug discovery applications. Therefore, to address the challenges in drug discovery domain, this paper introduces a two-stage sampling-based classifier drug discovery system. This system uses sampling strategies to study the class imbalance problem, to maximize the detection of potential drug-candidate conformations and recommend them to the biologists/pharmacologists during in vitro analysis. It also suggests the non-drug candidate protein conformations that could be discarded during the clinical trials, thus reducing the substantial investment of resources.

The novel contributions of this work are outlined as below:

- This research work studies the class imbalance problem in drug discovery applications, and how it affects the detection rates of potential drug-binding protein conformations.
- It introduces new ML strategies and presents a case study to study the effects of class imbalance problem, counter them, and maximize prediction rates of potential drug-binding molecular conformations for target proteins ADORA2A and OPRK1.
- This work is the first step toward the design of a new comprehensive drug discovery system, which optimizes the detection of both non-drug-binding protein conformations that could be discarded and potential drug-binding protein conformations that should be retained for further used in docking.
- The proposed drug discovery approaches can help with reducing the time and cost of drug discovery, which is particularly desirable in global pandemic situations.

The rest of the paper is organized as follows: background and related work is discussed in Section 2 followed by the proposed two-stage sampling based ML classification framework in Section 3. In Section 4, we experimentally demonstrate and validate the efficacy of the two-stage sampling based ML classification framework in identification of potential drug candidate protein conformations. Finally, we summarize the effectiveness of our proposed automated ML-based models for prediction of potential drug candidate protein conformational selection in Section 5.

## 2. Background and Related Work

### 2.1. Class Imbalance Problem

In real-world drug discovery applications, most of the biomedical datasets observed are imbalanced in nature. The training phase of a supervised classifier constitutes data-learning performed on the given unequal distribution of representative samples from the imbalanced dataset. Here, the classifier aims to achieve a good classification/prediction performance without any consideration for the data distribution of each class. Hence, the resultant decision-making process tends to be more biased towards the majority class (which in the present case would be protein conformations that will not bind drugs), thereby leading to the misclassification of minority class samples (which in the present case would be protein conformations that will bind the drugs) [9]. Thus, the penalty cost of misclassification of minority samples is higher than the majority samples for a class imbalance problem [10]. Especially in drug discovery application, the class imbalance problem is of great consequence, since it leads to a higher risk of discarding the smaller population of protein conformations that can successfully bind to drugs due to them being misclassified as non-drug-binding protein conformations. In drug discovery literature, ensemble learning techniques have been proposed as a solution to handle the within- and between-class imbalance problem for prediction of drug–target interaction [11]. The main difference between our approach and other approaches is that, in our work, we tackle the class imbalance problem and also introduce a new perspective to maximize the detection of both potential drug candidate conformations and non-drug candidate conformations via the proposed two-stage sampling-based classifier system. The remainder of this paper discusses more about the proposed method, design of the two-stage sampling-based classifier system, and its experimental efficacy.

### 2.2. Sampling Strategies

The two broad categories of sampling strategies often employed are: (1) Undersampling approach: where sampling is performed by removal of additional data instances from the majority class samples and (2) Oversampling approach: where sampling is performed by the addition of artificial data instances to the minority class samples [10]. One of the most popular undersampling techniques is a random undersampling approach, wherein *N* samples are selected at random from the majority class to be included as part of a new smaller training set that consists of equal distribution (same *N* number of samples) of minority class samples. The main drawback of the undersampling approach is that it entails a substantial risk of loss of important information due to elimination of a large amount of original data samples from the new training phase [10,12,13], whereas the oversampling approach has no loss of information involved since all the original data samples are retained in the training phase. The oversampling approach generates artificial data samples of the minority class and adds them to the new training dataset to obtain the desired well-balanced data, which consists of equal distribution of majority and minority class data samples [10]. A synthetic minority oversampling technique (SMOTE) proposed by Chawla [14] creates new synthetic data samples of the minority class and adds them to the new training data set, thus reducing the overfitting problem that arises due to data replication. For this, SMOTE identifies *K* nearest neighbors for each data sample belonging to the minority class and then performs regression to fit the data samples to a line in order to generate the new synthetic data samples for the minority class [14]. The only problem with oversampling approach is that, for larger datasets, the increase in training data size also implies a corresponding increase in time-complexity of the data-learning phase. In the remainder of this paper, we will discuss several existing supervised ML techniques and introduce our novel ML approaches that leverage the sampling strategies to alleviate the class imbalance problem and maximize detection of potential drug-candidate molecular conformations for proteins ADORA2A and OPRK1.

### 2.3. Datasets Description

In this work, proteins ADORA2A (Adenosine A2a Receptor) and OPRK1 (Opioid Receptor Kappa 1) are considered for case study and experimental validation of the proposed method. The conformations of these two proteins have been extensively characterized, and the protein conformations that (i) will bind ligands and (ii) will not bind ligands are known and have been documented and published [1]. The conformations of the proteins were collected from 0.6 microsecond molecular dynamics simulations using full protein, membrane, and hydration environments on the Moldyn High Performance Cluster, and the docking was performed on the Titan Supercomputer at the Oak Ridge National Laboratory in Oak Ridge, TN, USA and on the Newton Supercomputer at the University of Tennessee, using the VinaMPI docking engine as described in Evangelista et al. The protein conformations that were docking the protein ligands with a high score were identified as described in [1]. Each protein MD trajectory consisted of 3000 protein conformations.

ADORA2A: This gene encodes a member of the guanine nucleotide-binding protein (G protein)-coupled receptor (GPCR) superfamily, which is subdivided into classes and subtypes. The given dataset consists of 50 attributes with 3000 molecular conformations among which 850 conformations have the potential to bind with the protein and 2147 conformations do not bind with the protein. Here, the imbalance ratio is 3:1 i.e., or every datasample belonging to minority class, there are three data samples belonging to the majority class.

OPRK1: This gene encodes an opioid receptor, and is a member of the 7 transmembrane spanning GPCR family.This is a Protein Coding gene. The given dataset consists of 50 attributes with 2999 molecular conformations among which 137 conformations have the potential to bind with the protein and 2862 conformations do not bind with the protein. Here, the imbalance ratio is 20:1 i.e., for every datasample belonging to minority class, there are 20 datasamples belonging to the majority class.

For each of the conformations of the ADORA2A and OPRK1 proteins described in Table 1, the molecular descriptors were calculated using the protein descriptors of the program MOE [15]. These descriptors are used to quantify various physical and chemical properties of the protein conformations. These descriptors are mostly based on the whole protein conformations, i.e., they are not limited to the small part of the protein where the ligands do bind. These descriptors have been found useful to characterize proteins and their conformations in ways that can distinguish them from other proteins or conformations [16].

The goal is to identify the maximum number of molecular conformations related to the ADORA2A and OPRK1 proteins to recommend the identified smaller subset of potential drug candidate conformations for more efficient docking calculations.The datasets have an active binding feature as the “dependent variable” which has 2 classes labeled as either 0 or 1 to indicate the binding outcome. Class 0 describes that known ligands do not bind well to the given protein conformation, and class 1 conveys that known ligands bind well to the given protein conformation. In this work, we have explored seven supervised ML classification models: Methodology 1 classification using LR, Methodology 2 classification using GB and KNN, re-balance the dataset using SMOTE followed by Methodology 2 classification techniques: SMOTE-GB, and SMOTE-KNN, and decision fusion using LR+SMOTE-GB and LR+SMOTE-KNN techniques. Their performance evaluation was conducted on 10%, 20% and 30% of training data sizes, where the training samples were randomly chosen from the original dataset. This training set range was chosen because, in reality, there are only a smaller number of conformations available for data-learning and training the supervised classifier. The LR classifier hyperparameter was empirically chosen as C=5. In the context of protein conformational selection for drug discovery application, a confusion matrix presents the summary of prediction/classification results of a given protein conformation as belonging to one of the below four cases:

- True positives (TP): The cases for which the classifier predicted “Active Binding” (drug candidate) and the conformations were actually bounded to the target protein.
- True negatives (TN): The cases for which the classifier predicted “Inactive Binding” (non-drug candidate) and the conformations were actually not bounded to the target protein.
- False negatives (FN): The cases for which the classifier predicted “Inactive Binding” (non-drug candidate) but the conformations were actually bounded to the target protein.
- False positives (FP): The cases for which the classifier predicted “Active Binding” (drug candidate), but the conformations were actually not bounded to the target protein.

### 2.4. Logistic Regression

Logistic regression (LR) is one of the popular ML techniques that is used to perform predictive analysis of data, when the data are categorical or has binary classes. The goal of LR is to determine the best fitting model that describes the relationship between one dependent binary variable against a group of independent variables [17]. LR models the transformed probability as a linear relationship of independent variables. Let *y* be the binary outcome indicating failure/success (not belong to the class/belongs to the class) with 0/1, respectively, and *p* be the probability of y=1 as p=prob(y=1). Conversely, y=0 can be expressed as 1−p. Then, LR models the outcome *y* based on linear combination of the independent data variables $x_{1},x_{2},\ldots ,x_{k}$ and their respective parameter/weight values β_1_, β_2_, …, β_k_ via maximum likelihood method as given by:

(1) $$f\left(p\right)=\log\frac{p}{p-1}=\beta_{0}+\beta_{1}x_{1}+\beta_{2}x_{2}+\beta_{3}x_{3}+\ldots +\beta_{k}x_{k}$$

For an accurate LR model with minimum error, we determine the cost function J(θ) such that the square error between the actual values of *y* and its predicted values $\hat{y}$ is minimized. Thus, using mean squared error formulation for LR makes the cost function J(θ) non-convex with multiple local minima. Therefore, we use cross entropy based LR formulation, where cross entropy H is defined as the dissimilarity measure between the probability *p* corresponding to actual values *y* and predicted probability $\hat{p}$ corresponding to the predicted values $\hat{y}$ can be calculated as:

(2) $$\begin{array}{cc} H(p,\hat{p}) & =-\sum_{i}p_{i}\log\hat{p}\\ \; & =-y\log\hat{y}-\left(1-y\right)\log\left(1-\hat{y}\right) \end{array}$$

From Equation (2), the new cross entropy based LR cost function is formulated as:

(3) $$\begin{array}{cc} J(\theta ) & =-\frac{1}{N}\sum_{n=1}^{N}H\left(p,\hat{p}\right)\\ \; & =-\frac{1}{N}\sum_{n=1}^{N}\left[y\log\hat{y}+\left(1-y\right)\log\left(1-\hat{y}\right)\right]\; \end{array}$$

### 2.5. Gaussian Naive Bayes Classifier

Naive Bayes classifier is another commonly used ML supervised technique for data classification purposes. It follows the principle of maximum *a* posteriori probability (MAP) estimation for a probabilistic classification of data. Let y∈{0,1} be the binary class outcome for a given new data point *x*. According to Bayes theorem, the posterior probability P(y|x) can be expressed in terms of prior probability P(y) and likelihood P(y|x) as described by:

(4) P(y|x)=(P(x|y)∗P(y))/P(x)

where

- P(y|x) is the probability of outcome *y* given the data point *x*. This is called the posterior probability.
- P(x|y) is the probability of data point *x* given that the outcome *y* was true. This is called the likelihood.
- P(y) is the probability of outcome *y* being true across all of the data. This is called the prior probability of *y*.
- P(x) is the probability of the data averaged over all of the outcomes.

Therefore, the posterior probability for different classes can be calculated from Equation (4). The class with highest probability is chosen for classifying the new data point *x*. This strategy is called the MAP estimation as formulated below:

(5) max(P(y|x))=max(P(x|y)∗P(y))

Gaussian Naive Bayes (GB) classifier is used when the features have continuous values and follow Gaussian or normal distribution [18]. The likelihood of features is assumed to be Gaussian and is given by

(6) $$P\left(x|y\right)=\frac{1}{\sqrt{2\pi \sigma_{y}^{2}}}\exp(\frac{-{\left(x-\mu_{y}\right)}^{2}}{2\sigma_{y}^{2}})$$

where $\mu_{y}$ denotes the mean of class *y* and $\sigma_{y}^{2}$ gives the variance of class *y*. The standard normal distribution N(μ,σ) is a bell shaped density described by its mean μ=0 and standard deviation σ=1.

### 2.6. K-Nearest Neighbor Classifier

K-nearest neighbor (KNN) classifier is a widely used ML technique for its easy interpretation and low calculation time for both classification and regression problems. Given a new data point *x* which has to be assigned to one of the classes y∈{0,1}, the KNN algorithm performs the classification task as described by the following steps [19]:

- Choose the *K* neighbors.
- Calculate the *K* nearest neighbors $y_{i}$ of the new data point *x*: $D\left(x,y_{i}\right)=\sqrt{{\left(x-y_{i}\right)}^{2}}$ where Euclidean distance is used as a metric to determine the “nearness” or “closeness” criteria.
- Choose the *K* nearest neighbors of *x* in accordance to the minimum Euclidean distance between *x* and its *K* closest neighbors as defined by $\min D(x,y_{i})$.
- Among these chosen *K* neighbors, count the number of data points in each category of class.
- Assign the new data point *x* to the category where the neighborhood count is maximum.

### 2.7. SMOTE Algorithm

SMOTE is a popular oversampling technique which creates new synthetic data points rather than by oversampling with replacement or redundant samples of the minority class data [9]. SMOTE algorithm is detailed below [12,14,20]:

- Choose *K* neighbors.
- The minority class is oversampled by taking the difference between each minority class sample (feature vector) and its *K* nearest neighbors.
- Multiply this difference by a random weight between 0 and 1, and add it to the feature vector. This causes the selection of a random point along the line-of-fit model between the two specific features.
- Thus, the synthetic data points of minority class samples are created for the desired well-balanced dataset.

## 3. Proposed Methodologies

Among several classifiers applied to the training phase of the dataset, it was experimentally found that LR had excellent classification performance in detection of maximum non-drug conformations and GB classifier provided better detection of potential binding drug candidate conformations compared to other classifiers from our previous work [21]. For classification purposes, we denote the non-drug molecular conformation samples that have inactive binding to target proteins as belonging to class 0 and potential drug candidate molecular conformations that have active binding to target proteins as class 1 samples. Thus, this is a binary classification problem. We use this as a basis to design our comprehensive drug-candidate conformation detection system that leverages the detection of both potential drug- and non-drug conformations for expedited clinical trials. This system comprises of two stages:

- Methodology 1: This first stage is responsible for maximizing the detection of non-drug conformations (class 0 samples) using an LR classifier.
- Methodology 2: The second stage comprises of a classification system using GB classifier (GB) and KNN classifier (KNN). They are responsible for maximizing the detection of potential binding drug candidate conformations/class 1 samples.

### Two-Stage Sampling-Based Classifier Algorithm

**Figure 1:**
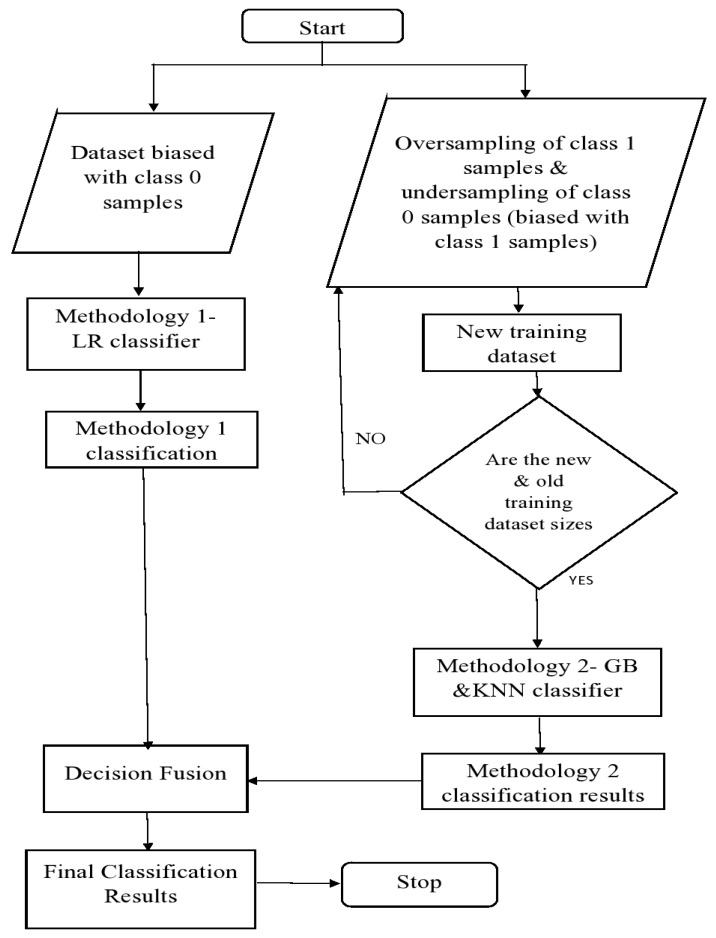
Flowchart of the proposed two-stage sampling-based classifier.

- Initially, methodology 1 is applied to our original training data which inherently has a biased population that consists of a large number inactive binding non-drug conformations/class 0 samples. Both the identified class 0 and class 1 samples are recorded as methodology 1 results.
- A new training dataset is obtained by random undersampling of class 0 samples and oversampling of desired class 1 samples using the SMOTE algorithm. This process tackles the class-imbalance problem and maximizes the detection rate of active binding drug candidate conformations/class 1 samples. For consistency, the size of new training dataset is kept the same as the size of original training dataset. Hence, there is no increase in the data-learning time and the effect of overfitting is reduced because of the SMOTE technique.
- Methodology 2 classification system is then applied to our new training dataset which is biased with class 1 samples. We denote the corresponding technique of generating a new balanced dataset using SMOTE followed by the methodology 2 classification system as SMOTE-GB and SMOTE-KNN techniques. This step of methodology 2 classification is performed to reaffirm the active binding and inactive binding molecular conformations that are identified by methodology 1 for a more robust drug-candidate conformation detection system. Since methodology 2 has greater affinity towards detection of potential active binding drug-candidate conformations, the new unique class 1 samples identified are recorded in methodology 2 results.
- Finally, a decision fusion strategy via majority voting is implemented to uniquely identify the total number of potential active binding drug candidate- and inactive binding non-drug molecular conformations acquired from methodology 1 and methodology 2 classification models. The corresponding decision fusion strategies are denoted by LR+SMOTE-GB and LR+SMOTE-KNN techniques. Figure 1 depicts a flowchart of the proposed two-stage tailored drug conformation classification system.

The motivation behind using methodology 1 is to identify the maximum number of non-drug protein conformations, whereas methodology 2 is used to provide an additional layer of security to reconfirm the potential-drug conformations identified by the methodology 1 along with the identification of new potential-drug conformations through decision fusion strategy. This form of two-stage tailored binary classification system ensures maximum detection of both potential drug candidate- and non-drug protein conformations at each step. Consequently, it aims to reduce the failure rates incurred during the clinical trials involved in the drug development process. The identified protein conformations can considered by the clinicians for informed decision-making to discard the non-drug candidate protein conformations and retain only the potential drug-candidate protein conformations for in vitro experimental approaches, thus reducing the substantial investment of resources that include time, cost, and rigorous lab testing trials.

## 4. Results and Discussion

### 4.1. Datasets Description

In our initial experiments, several ML classifiers such as support vector machines (SVMs), GB, LR, and KNN classifiers were explored. Most of them yielded a good classification performance with a high detection of non-drug conformations/TNs, but a very low detection of drug candidate conformations/TPs. This was mainly because of the class imbalance problem, due to which the performance of ML classification models was more biased towards the majority class; (i.e., class 0 samples). This prompted us to design a system specifically to tackle the class imbalance problem and maximize the detection of TPs and TNs, and evaluate the performance of our proposed methods through the standard classification metrics that adequately represent the model performance.

### 4.2. Performance Evaluation

This section comprises of three test cases, namely, Case 1: Training set with biased population of class 0 samples. Case 2: Training set with biased population of class 1 samples. Finally Case 3: Decision fusion strategy. The quantitative performance analysis of the proposed classification techniques for each of the test cases are conducted using the measures as defined below:

Classification Accuracy (Acc): The accuracy of a test is defined as the ability to differentiate the “Active Binding” and “Inactive Binding” cases correctly. Mathematically, it is given by

$$\mathrm{Classification}\;\;\;\mathrm{Accuracy}=\frac{TP+TN}{N}\times 100$$

where

N=TP+TN+FP+FN

Sensitivity (Se): The sensitivity of a test is defined as the ability to determine the “Active Binding” cases correctly. Mathematically, it is given by

$$\mathrm{Sensitivity}=\frac{TP}{TP+FN}$$

Specificity (Sp): The specificity of a test is defined as the ability to determine the “Inactive Binding” cases correctly. Mathematically, it is given by

$$\mathrm{Specificity}=\frac{TN}{TN+FP}$$

Performance Evaluation using AUC-ROC Curve: ROC curve (or receiver operating characteristic curve) is a plot that summarizes the performance of a binary classification model on the positive class and the area under the curve (AUC) represents degree or measure of separability. It measures the capability of the model to distinguish between the classes. AUC can be calculated to give a single score for a classifier model across all threshold values. Higher AUC represents better prediction or classification capabilities of the model. The ROC curve is plotted with True Positive Rate (TPR) against the False Positive Rate(FPR) where TPR is on *y*-axis and FPR is on the *x*-axis.

### 4.3. Computational Evaluation on the ADORA2A Dataset

Case 1: Training set biased population of class 0 samples: Table 2 gives the per-class samples present in accordance with the various training sizes considered.

From Table 3, it can be noted that LR identified 151 TPs from 579 samples of class 1 and 1313 TNs from 1519 samples of class 0 for a training size of 30%. Thus, the observed results across all cases validate our argument that LR has the best classification performance for detection of TNs or non-drug conformations.

From Table 4, it can be seen that GB identified 279 TPs from 579 samples of class 1 and 1059 TNs from 1519 samples of class 0. Similarly, KNN identified 149 TPs from 579 samples of class 1 and 1245 TNs from 1519 samples of class 0 for training size of 30%. Thus, it can be noted that overall GB had the best classification performance in methodology 2 and gave better detection rates of TPs or potential drug candidate conformations over the KNN classifier.

Case 2: New training set with biased population of class 1 samples: This case studies the effects of induced class imbalance on the prediction rates of molecular conformations. The goal here is to maximize the detection of TPs identified by methodology 2 via the two stage sampling based approach in order to improve the model performance. For this, we perform SMOTE oversampling of class 1 samples and undersampling of class 0 samples, such that the new training data size is consistent with the original training data size. Methodology 2 is then employed for data-learning on the resultant new training set. Table 5 gives the per-class distribution in the new training dataset. From Table 5 results, we observe that the size of the new training dataset is the same as that of the original training dataset. For instance, the original training dataset had 628 class 0 samples and 271 class 1 samples for training size of 30%. The new training dataset has the same number of class 0 samples = 271 and number of class 1 samples = 628 for 30% training size.

Table 6 describes the overall improved classification performance of the proposed two-stage classification system and the effects of induced class imbalance of class 1 data population. From Table 6, SMOTE-GB identified 384 TPs, which is more than the 279 TPs identified using the original training dataset in Table 4 for training size of 30%. In particular, the SMOTE-KNN classifier had the best methodology 2 classification performance, wherein it identified 504 TPs, which is more than the 149 TPs identified using the original training dataset in Table 4 for training size of 30%. The affable effects of induced class imbalance are evident from the boost in classification performance of methodology 2 techniques. In particular, the SMOTE-KNN technique works on the concept of nearest neighbor algorithm and more induced neighbors via oversampling of class 1 samples implies better statistical conclusion of the underlying population distribution of the potential drug candidate conformations.

Table 7 describes the overall TPs and TNs reconfirmed and identified, including the unique TPs identified by Methodology 2. For the training size of 30%, SMOTE-GB reconfirmed on 134 TPs identified by methodology 1 apart from identifying 250 new TPs. Thus, in total, it identified 384 TPs using the new training dataset. The SMOTE-KNN classifier reconfirmed the 140 TPs identified by methodology 1 classifier aside from identifying 364 new TPs. Thus, in total, 504 TPs were identified using the new training dataset. Thereby, Table 7 further demonstrates the effectiveness of the proposed two-stage sampling-based classification approach.

Case 3: Decision Fusion: This case explores a decision fusion strategy via a majority voting scheme as a means to assimilate the classification conclusions from methodology 1—LR with the results from the methodology 2 system: SMOTE-GB and SMOTE-KNN techniques to arrive at a global consensus of the overall identified molecular conformations that possess active binding property. The quantitative performance of proposed decision fusion method is determined in terms of the below measures:

$$\mathrm{Total}\;\mathrm{accuracy}=\frac{TN_{1}+TP_{1}+New\;TN_{2}+New\;TP_{2}}{N_{test}}$$

where $N_{test}$ represents the total number of class 1 and class 0 samples in the test set.

$$\mathrm{Total}\;TP\;\mathrm{accuracy}\;\left(TP_{acc}\right)=\frac{TP_{1}+New\;TP_{2}}{N_{test}^{1}}$$

where $N_{test}^{1}$ represents the total number of class 1 samples in the test set.

$$\mathrm{Total}\;TN\;\mathrm{accuracy}\;\left(TN_{acc}\right)=\frac{TN_{1}+New\;TN_{2}}{N_{test}^{0}}$$

where $N_{test}^{0}$ represents the total number of class 0 samples in the test set.

**Figure 2:**
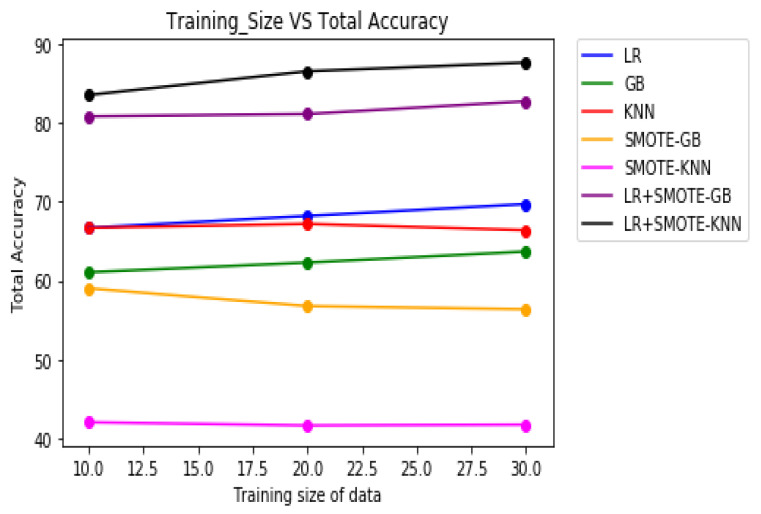
Plot of overall classification accuracies across varying training sizes for case 1, case 2, and decision fusion for protein ADORA2A.

**Figure 3:**
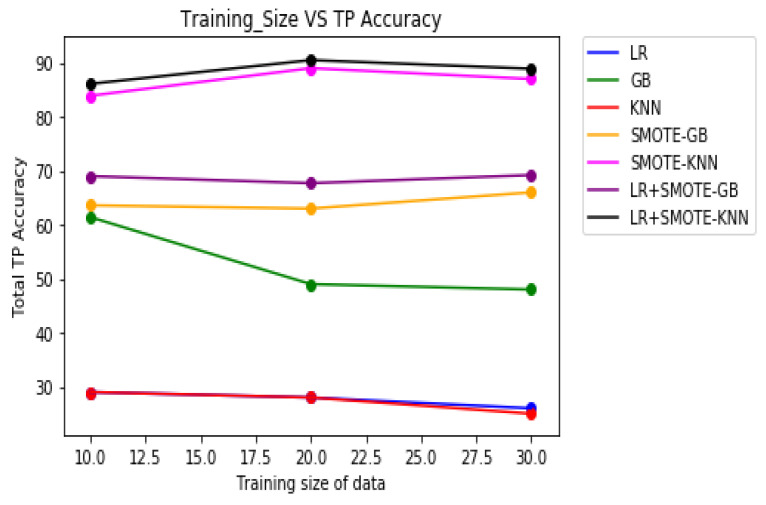
Plot of TP accuracies across varying training sizes for case 1, case 2, and decision fusion for protein ADORA2A.

**Figure 4:**
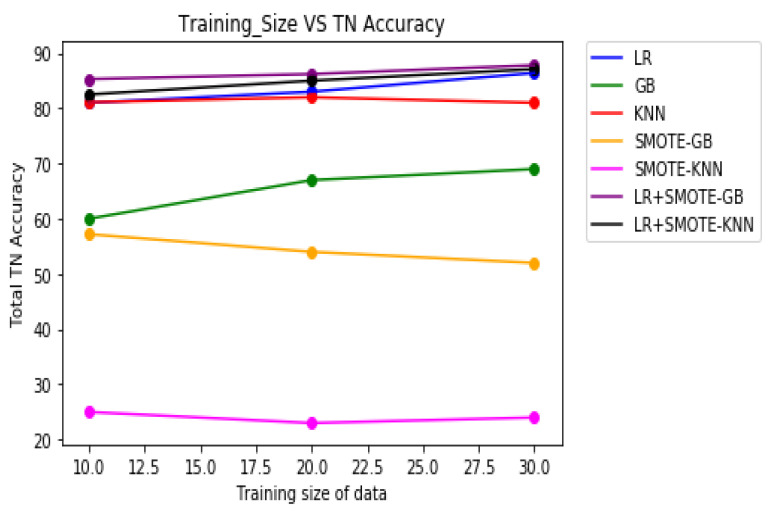
Plot of TN accuracies across varying training sizes for case 1, case 2, and decision fusion for protein ADORA2A.

Table 8 demonstrates the overall classification performance of the proposed decision fusion case. From Table 8 and Figure 2, Figure 3 and Figure 4, it can be inferred that the overall classification performance of the proposed methods: LR+SMOTE-GB and LR+SMOTE-KNN techniques were significantly superior to individual classification performance of LR, GB, and KNN classifiers as given in Table 3 and Table 4. From Figure 2, Figure 3 and Figure 4, especially, LR+SMOTE-KNN had excellent classification performance for identification of the molecular conformations for protein ADORA2A in terms of overall classification accuracy and TP accuracy measures.

### 4.4. Computational Evaluation on the OPRK1 Dataset

Case 1: Training set biased population of class 0 samples: Table 9 gives the per-class distribution in accordance with the various training sizes considered.

From Table 10, the observed results across all cases validate our argument that LR has the best classification performance for detection of TNs or non-drug conformations due to the class imbalance problem.

From Table 11, it can be seen that GB identified 51 TPs from 96 samples of class 1 and 804 TNs from 2004 samples of class 0 for a training size of 30%. Similarly, KNN could not identify any TPs from 96 samples of class 1 but identified 100% of TNs of class 0 samples for a training size of 30% due to the class imbalance problem. Thus, it can be noted that overall GB had the best classification performance as in Methodology 2 with better detection rates of TPs or potential drug candidate conformations over the KNN classifier.

Case 2: New training set with biased population of class 1 samples: Table 12 gives the per-class distribution in the new training dataset. From Table 12, it can be noted that the size of new and original training datasets are the same. From Table 13, SMOTE-GB identified 61 TPs and SMOTE-KNN identified 93 TPs, which is more than the TPs identified using the original training dataset in Table 11 for the training size of 30%. The effects of induced class imbalance are evident from the boost in classification performance of both of the methodology 2 classifiers. From Table 14 for the training size of 30%, methodology 1 could not identify any TP, but methodology 2 classifiers—SMOTE-GB identified 61 new TPs and the SMOTE-KNN classifier identified 93 new TPs.

**Figure 5:**
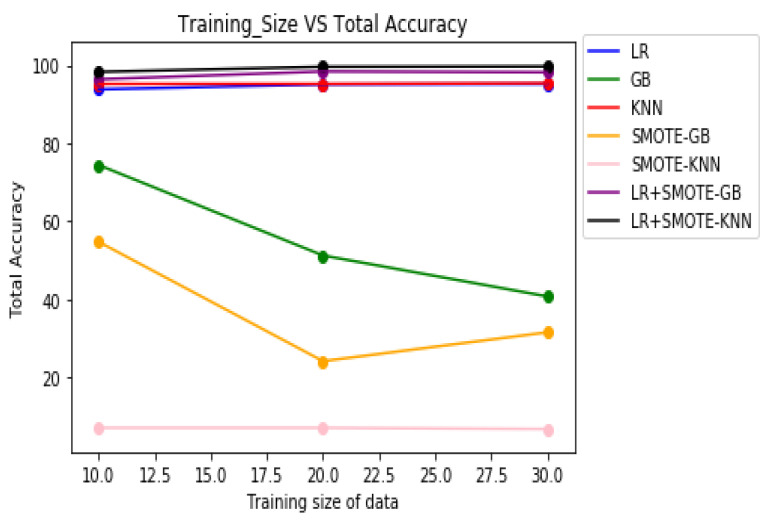
Plot of overall classification accuracies across varying training sizes for case 1, case 2, and decision fusion for protein OPRK1.

**Figure 6:**
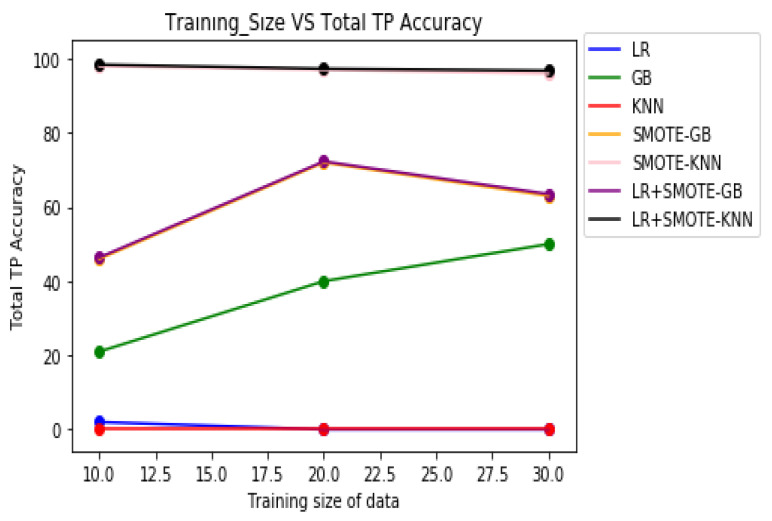
Plot of TP accuracies across varying training sizes for case 1, case 2, and decision fusion for protein OPRK1.

**Figure 7:**
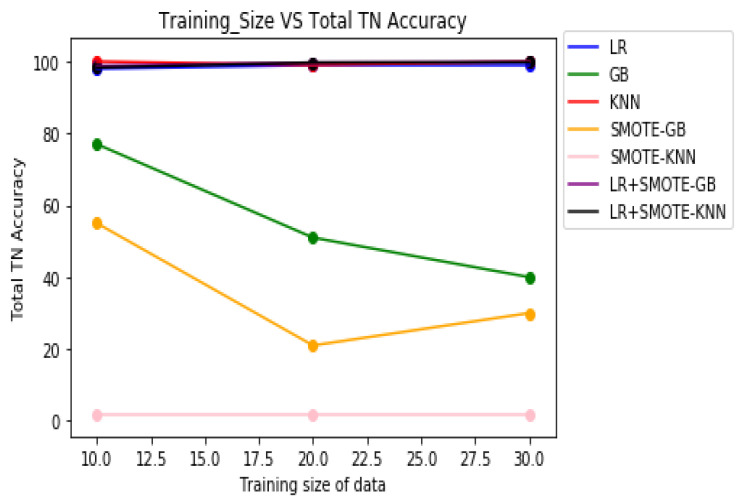
Plot of TN accuracies across varying training sizes for case 1, case 2, and decision fusion for protein OPRK1.

Case 3: Decision Fusion: Table 15 demonstrates the overall classification performance of the proposed decision fusion case. From Table 15 and Figure 5, Figure 6 and Figure 7, LR+SMOTE-KNN had excellent classification performance for identification of the molecular conformations for protein OPRK1 in terms of overall classification accuracy and TP accuracy measures. Therefore, it can be surmised that this work presents a new avenue that successfully leverages the class imbalance problem to design a novel and effective drug conformation detection system for efficient clinical trials in drug discovery application.

**Figure 8:**
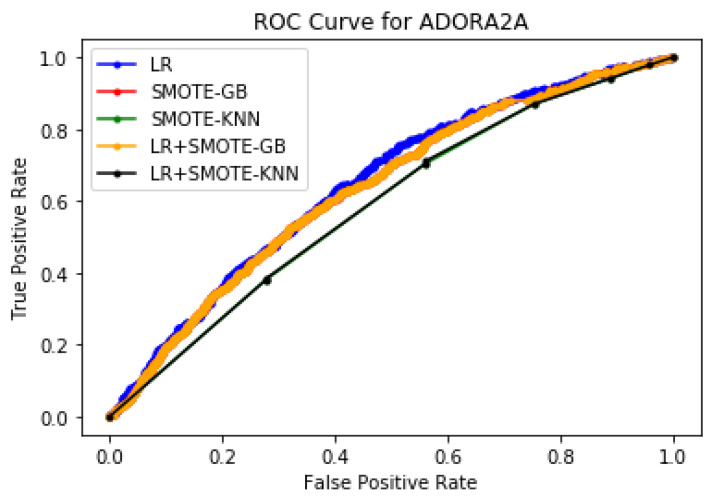
Plot of ROC curve for ADORA2A protein for a training size of 30%.

**Figure 9:**
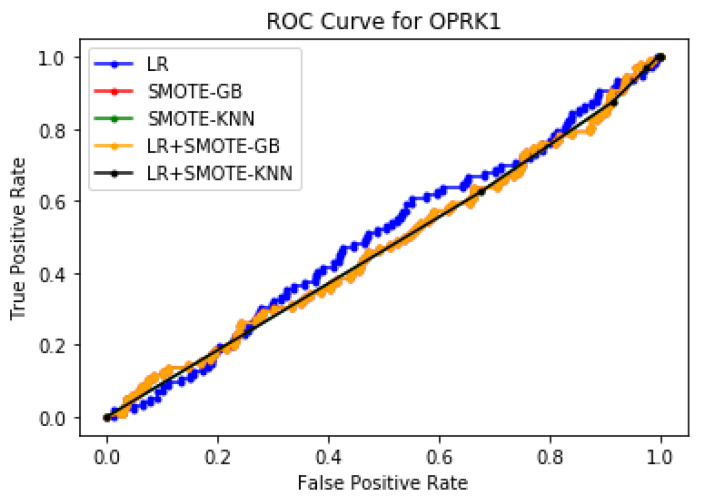
Plot of ROC curve for OPRK1 protein for a training size of 30%.

Performance evaluation using ROC curves, AUC, and F1 scores: To further quantitatively validate the efficacy of our proposed methodologies, statistical measures such as ROC curves, F1, and AUC scores were computed. Figure 8 presents the ROC curves and Table 16 gives the AUC scores and F1 scores for the proposed ML methodologies for protein ADORA2A. It can be noted that the proposed LR+SMOTE-GB approach has the best performance in terms of AUC score = 0.638, whereas the other proposed method LR+SMOTE-KNN had better F1 score = 0.792 for protein ADORA2A. Similarly, Figure 9 illustrates the ROC curves for the proposed ML methodologies and Table 17 gives the AUC scores obtained for protein OPRK1. It can be noted that both LR+SMOTE-GB and LR+SMOTE-KNN had performed well and has the best area under the curve with an AUC score of 0.475 and 0.470 respectively. Similarly, the proposed method LR+SMOTE-KNN had the best F1 score = 0.968 than the LR+SMOTE-GB method with F1 score = 0.770. These results corroborate our argument that it is highly beneficial to look into a comprehensive drug discovery system that maximizes detection of both the potential drug and non-drug protein conformations for efficient drug discovery process.

## 5. Conclusions

In this paper, we introduced a novel two-stage sampling-based ML classification techniques to overcome the class imbalance problem and maximize the prediction of non-drug-binding and potential drug-binding molecular conformations for the target proteins ADORA2A and OPRK1. The motivation of this work is to accelerate the drug discovery process. Applied to novel protein targets, this approach will lead to reducing of the failure rates in clinical trials for an efficient drug development process, as it will allow for identifying the drug-binding conformations of frequent off-target proteins. It was experimentally evaluated that the proposed decision fusion techniques: LR+SMOTE-GB and LR+SMOTE-KNN demonstrated superior classification performance compared to the individual classifier ML models: LR, GB, and KNN. In particular, LR+SMOTE-KNN outperformed other methods and gave the maximum prediction rate of molecular conformations for proteins ADORA2A and OPRK1 with a total accuracy of 87.6% and 99.7% for training size 30%. This work further highlights the need and advantages of ML techniques to hasten the drug discovery process.

## 6. Future Scope

Random undersampling could lead to a high probability of important information loss pertinent to non-drug molecular conformations. Hence, as a future endeavor, this work will be a foundation for improved drug discovery system design that will preserve the information present in both potential drug- and non-drug conformations.

## Figures and Tables

**Table 1:** Protein descriptors for ADORA2A and OPRK1 datasets.

Protein Property	Description
pro_mass	Protein Mass
pro_pI_3D	Structure-based pI Prediction
pro_coeff_fric	Frictional Coefficient
pro_coeff_diff	Diffusion coefficient
pro_r_gyr	Radius of Gyration
pro_r_solv	Hydrodynamic Radius
pro_sed_const	Sedimentation Constant
pro_eccen	Protein Eccentricity
pro_asa_vdw	Water Accessible Surface Area
pro_asa_hyd	Hydrophobic Surface Area
pro_asa_hph	Hydrophilic Surface Area
pro_volume	Protein Volume
pro_mobility	Protein Mobility
pro_helicity	Protein Helix Ratio
pro_henry	Henry’s Function f(ka)
pro_net_charge	Protein Net Charge
pro_app_charge	Protein Charge at Debye Length
pro_dipole_moment	Protein Dipole Moment
pro_hyd_moment	Hydrophobicity moment
pro_zeta	Zeta Potential
pro_zdipole	Zeta Dipole Moment
pro_zquadrupole	Zeta Quadrupole Moment
pro_patch_hyd	Area of hydrophobic protein patch(es)
pro_patch_hyd_1	Area of largest hydrophobic protein patch(es)
pro_patch_hyd_2	Area of 2 largest hydrophobic protein patch(es)
pro_patch_hyd_3	Area of 3 largest hydrophobic protein patch(es)
pro_patch_hyd_4	Area of 4 largest hydrophobic protein patch(es)
pro_patch_hyd_5	Area of 5 largest hydrophobic protein patch(es)
pro_patch_hyd_n	Count of hydrophobic protein patch(es)
pro_patch_ion	Area of ionic protein patch(es)
pro_patch_ion_1	Area of largest ionic protein patch(es)
pro_patch_ion_2	Area of 2 largest ionic protein patch(es)
pro_patch_ion_3	Area of 3 largest ionic protein patch(es)
pro_patch_ion_4	Area of 4 largest ionic protein patch(es)
pro_patch_ion_5	Area of 5 largest ionic protein patch(es)
pro_patch_ion_n	Count of ionic protein patch(es)
pro_patch_neg	Area of negative protein patch(es)
pro_patch_neg_1	Area of largest negative protein patch(es)
pro_patch_neg_2	Area of 2 largest negative protein patch(es)
pro_patch_neg_3	Area of 3 largest negative protein patch(es)
pro_patch_neg_4	Area of 4 largest negative protein patch(es)
pro_patch_neg_5	Area of 5 largest negative protein patch(es)
pro_patch_neg_n	Count of negative protein patch(es)
pro_patch_pos	Area of positive protein patch(es)
pro_patch_pos_1	Area of largest positive protein patch(es)
pro_patch_pos_2	Area of 2 largest positive protein patch(es)
pro_patch_pos_3	Area of 3 largest positive protein patch(es)
pro_patch_pos_4	Area of 4 largest positive protein patch(es)
pro_patch_pos_5	Area of 5 largest positive protein patch(es)
pro_patch_pos_n	Count of positive protein patch(es)

**Table 2:** Number of class 0 samples and class 1 samples in the original training dataset.

Training	Class 0 Samples		Class 1 Samples	
Size	Training	Testing	Training	Testing
10	202	1945	97	753
20	413	1734	186	664
30	628	1519	271	579

**Table 3:** Classification performance of Methodology 1-LR on the original training dataset.

Training	Methodology 1 Classifier—LR						
Size	${\mathrm{TP}}_{1}$	${\mathrm{TN}}_{1}$	${\mathrm{FN}}_{1}$	${\mathrm{FP}}_{1}$	Acc	Se	Sp
10	222	1577	531	368	66.7	0.29	0.81
20	188	1449	476	285	68.2	0.28	0.83
30	151	1313	428	206	69.7	0.26	0.864

**Table 4:** Classification performance of Methodology 2—GB and KNN on the original training dataset.

Training	Methodology 2—GB						
Size	${\mathrm{TP}}_{2}$	${\mathrm{TN}}_{2}$	${\mathrm{FN}}_{2}$	${\mathrm{FP}}_{2}$	Acc	Se	Sp
10	463	1185	290	760	61.08	0.614	0.6
20	331	1165	333	569	62.3	0.49	0.67
30	279	1059	300	460	63.7	0.48	0.69
**Training**	**Methodology 2—KNN**						
**Size**	${\mathrm{TP}}_{2}$	${\mathrm{TN}}_{2}$	${\mathrm{FN}}_{2}$	${\mathrm{FP}}_{2}$	Acc	Se	Sp
10	224	1578	529	367	66.7	0.29	0.81
20	188	1424	476	310	67.2	0.28	0.82
30	149	1245	430	274	66.4	0.25	0.81

**Table 5:** Number of class 0 samples and class 1 samples in the new training dataset.

Training	Class 0 Samples		Class 1 Samples	
Size	Training	Testing	Training	Testing
10	97	1945	202	753
20	186	1734	413	664
30	271	1519	628	579

**Table 6:** Classification performance of SMOTE-GB and SMOTE-KNN on the new training dataset.

Training	Methodology 2 Classifier—SMOTE-GB						
Size	${\mathrm{TP}}_{2}$	${\mathrm{TN}}_{2}$	${\mathrm{FN}}_{2}$	${\mathrm{FP}}_{2}$	Acc	Se	Sp
10	479	1114	274	831	59.04	0.636	0.572
20	420	943	244	791	56.8	0.63	0.54
30	384	800	195	719	56.4	0.66	0.52
**Training**	**Methodology 2 Classifier—SMOTE-KNN**						
**Size**	${\mathrm{TP}}_{2}$	${\mathrm{TN}}_{2}$	${\mathrm{FN}}_{2}$	${\mathrm{FP}}_{2}$	Acc	Se	Sp
10	632	504	121	1441	42.1	0.839	0.25
20	592	408	72	408	41.7	0.89	0.23
30	504	373	75	1146	41.8	0.87	0.24

**Table 7:** Reconfirmation and identification of new TP by Methodology 2—SMOTE-GB and SMOTE-KNN.

Training	Methodology 2—SMOTE-GB		
Size	New ${\mathrm{TP}}_{2}$	New ${\mathrm{TN}}_{2}$	${\mathrm{TP}}_{2}$ Reconfirmed
10	298	84	181
20	262	46	158
30	250	22	134
**Training**	**Methodology 2—SMOTE-KNN**		
**Size**	New ${\mathrm{TP}}_{2}$	New ${\mathrm{TN}}_{2}$	${\mathrm{TP}}_{2}$ Reconfirmed
10	427	28	205
20	413	26	179
30	364	11	140

**Table 8:** Decision fusion of Methodology 1 and Methodology 2: LR+SMOTE-GB and LR+SMOTE-KNN.

Decision Fusion: LR+SMOTE-GB			
%Training Size	Total Accuracy	${\mathrm{TP}}_{\mathrm{acc}}$	${\mathrm{TN}}_{\mathrm{acc}}$
10	80.8	69	85.3
20	81.1	67.7	86.2
30	82.7	69.2	87.8
**Decision Fusion: LR+SMOTE-KNN**			
**%Training Size**	**Total Accuracy**	${\mathrm{TP}}_{\mathrm{acc}}$	${\mathrm{TN}}_{\mathrm{acc}}$
10	83.5	86.1	82.5
20	86.5	90.5	85
30	87.6	88.9	87.1

**Table 9:** Number of class 0 samples and class 1 samples in the original training dataset.

Training	Class 0 Samples		Class 1 Samples	
Size	Training	Testing	Training	Testing
10	289	2573	10	127
20	574	2288	25	112
30	858	2004	41	96

**Table 10:** Classification performance of Methodology 1-LR on the original training dataset.

Training	Methodology1 Classifier—LR						
Size	${\mathrm{TP}}_{1}$	${\mathrm{TN}}_{1}$	${\mathrm{FN}}_{1}$	${\mathrm{FP}}_{1}$	Acc	Se	Sp
10	3	2531	124	42	93.8	0.02	0.98
20	1	2282	111	6	95.1	0	0.99
30	0	2001	96	3	95.2	0	0.99

**Table 11:** Classification performance of Methodology 2—GB and KNN on the original training dataset.

Training	Methodology 2- GB						
Size	${\mathrm{TP}}_{2}$	${\mathrm{TN}}_{2}$	${\mathrm{FN}}_{2}$	${\mathrm{FP}}_{2}$	Acc	Se	Sp
10	27	1983	100	590	74.44	0.21	0.77
20	45	1186	67	1102	51.2	0.4	0.51
30	51	804	45	1200	40.7	0.5	0.40
**Training**	**Methodology 2- KNN**						
**Size**	${\mathrm{TP}}_{2}$	${\mathrm{TN}}_{2}$	${\mathrm{FN}}_{2}$	${\mathrm{FP}}_{2}$	Acc	Se	Sp
10	0	2573	127	0	95.2	0	1
20	0	2287	112	1	95.2	0	0.99
30	0	2004	96	0	95.4	0	1

**Table 12:** Number of class 0 samples and class 1 samples in the new training dataset.

Training	Class 0 Samples		Class 1 Samples	
Size	Training	Testing	Training	Testing
10	41	2004	858	96
20	25	2288	574	112
30	41	2004	858	96

**Table 13:** Classification performance of SMOTE-GB and SMOTE-KNN on the new training dataset.

Training	Methodology 2 Classifier—SMOTE-GB						
Size	${\mathrm{TP}}_{2}$	${\mathrm{TN}}_{2}$	${\mathrm{FN}}_{2}$	${\mathrm{FP}}_{2}$	Acc	Se	Sp
10	59	1420	68	1153	54.7	0.46	0.55
20	81	498	31	1790	24.1	0.72	0.21
30	61	602	35	1402	31.5	0.63	0.30
**Training**	**Methodology 2 Classifier—SMOTE-KNN**						
**Size**	${\mathrm{TP}}_{2}$	${\mathrm{TN}}_{2}$	${\mathrm{FN}}_{2}$	${\mathrm{FP}}_{2}$	Acc	Se	Sp
10	125	68	2	2505	7	0.98	0.02
20	109	60	3	2228	7	0.97	0.02
30	93	48	3	1956	6.7	0.96	0.02

**Table 14:** Reconfirmation and identification of new TP by Methodology 2—SMOTE-GB and SMOTE-KNN.

Training	Methodology 2—SMOTE-GB		
Size	*New ${\mathrm{TP}}_{2}$*	*New ${\mathrm{TN}}_{2}$*	${\mathrm{TP}}_{2}$ Reconfirmed
10	56	13	3
20	80	0	1
30	61	2	0
**Training**	**Methodology 2—SMOTE-KNN**		
**Size**	*New ${\mathrm{TP}}_{2}$*	*New ${\mathrm{TN}}_{2}$*	${\mathrm{TP}}_{2}$ **Reconfirmed**
10	122	0	3
20	108	1	1
30	93	0	0

**Table 15:** Decision fusion of Methodology 1 and Methodology 2: LR+SMOTE-GB and LR+SMOTE-KNN.

Decision Fusion: LR+SMOTE-GB			
%Training Size	Total Accuracy	${\mathrm{TP}}_{\mathrm{acc}}$	${\mathrm{TN}}_{\mathrm{acc}}$
10	96.4	46.4	98.8
20	98.4	72.3	99.7
30	98.2	63.5	99.9
**Decision Fusion: LR+SMOTE-KNN**			
**%Training Size**	**Total Accuracy**	${\mathrm{TP}}_{\mathrm{acc}}$	${\mathrm{TN}}_{\mathrm{acc}}$
10	98.3	98.4	98.3
20	99.6	97.3	99.7
30	99.7	96.8	99.8

**Table 16:** AUC Score and F1 Score of the proposed ML methodologies for ADORA2A.

Proposed ML Methodologies	AUC Score	F1 Score
LR	0.649	0.323
SMOTE-GB	0.638	0.456
SMOTE-KNN	0.588	0.452
LR+SMOTE-GB	0.638	0.684
LR+SMOTE-KNN	0.590	0.792

**Table 17:** AUC Score and F1 score of the proposed ML methodologies for OPRK1.

Proposed ML Methodologies	AUC Score	F1 Score
LR	0.496	0.030
SMOTE-GB	0.475	0.078
SMOTE-KNN	0.470	0.086
LR+SMOTE-GB	0.475	0.770
LR+SMOTE-KNN	0.470	0.968

## Data Availability

Statistical and computational models used are fully detailed in the main text. Data will be made available upon personal requests to authors.
